# A Comprehensive Microarray-Based DNA Methylation Study of 367 Hematological Neoplasms

**DOI:** 10.1371/journal.pone.0006986

**Published:** 2009-09-11

**Authors:** Jose I. Martin-Subero, Ole Ammerpohl, Marina Bibikova, Eliza Wickham-Garcia, Xabier Agirre, Sara Alvarez, Monika Brüggemann, Stefanie Bug, Maria J. Calasanz, Martina Deckert, Martin Dreyling, Ming Q. Du, Jan Dürig, Martin J. S. Dyer, Jian-Bing Fan, Stefan Gesk, Martin-Leo Hansmann, Lana Harder, Sylvia Hartmann, Wolfram Klapper, Ralf Küppers, Manuel Montesinos-Rongen, Inga Nagel, Christiane Pott, Julia Richter, José Román-Gómez, Marc Seifert, Harald Stein, Javier Suela, Lorenz Trümper, Inga Vater, Felipe Prosper, Claudia Haferlach, Juan Cruz Cigudosa, Reiner Siebert

**Affiliations:** 1 Institute of Human Genetics, Christian-Albrechts University, Kiel, Germany; 2 Cancer Epigenetics and Biology Program, Bellvitge Institute for Biomedical Research-Catalan Institute of Oncology, Barcelona, Spain; 3 Illumina, Inc., San Diego, California, United States of America; 4 Division of Cancer and Area of Cell Therapy and Hematology Service, Universidad de Navarra, Pamplona, Spain; 5 Molecular Cytogenetics Group, Centro Nacional Investigaciones Oncologicas, Madrid, Spain; 6 Second Medical Department, Christian-Albrechts University, Kiel, Germany; 7 Department of Genetics, University of Navarra, Pamplona, Spain; 8 Department of Neuropathology, University Hospital of Cologne, Cologne, Germany; 9 Department of Medicine III, University Hospital Grosshadern, Munich, Germany; 10 Department of Pathology, University of Cambridge, Cambridge, United Kingdom; 11 Department of Hematology, University of Duisburg-Essen, Essen, Germany; 12 Toxicology Unit, University of Leicester, Leicester, United Kingdom; 13 Institute of Pathology, University Hospital of Frankfurt, Frankfurt, Germany; 14 Institute of Pathology, Christian-Albrechts University, Kiel, Germany; 15 Institute of Cell Biology, University of Duisburg-Essen, Essen, Germany; 16 Reina Sofia Hospital, Instituto Maimonides de Investigación Biomédica de Córdoba, Cordoba, Spain; 17 Institute of Pathology, Campus Benjamin Franklin, Berlin, Germany; 18 Department of Hematology and Oncology, Georg-August University of Göttingen, Göttingen, Germany; 19 Munich Leukemia Laboratory (MLL), Munich, Germany; Health Canada, Canada

## Abstract

**Background:**

Alterations in the DNA methylation pattern are a hallmark of leukemias and lymphomas. However, most epigenetic studies in hematologic neoplasms (HNs) have focused either on the analysis of few candidate genes or many genes and few HN entities, and comprehensive studies are required.

**Methodology/Principal Findings:**

Here, we report for the first time a microarray-based DNA methylation study of 767 genes in 367 HNs diagnosed with 16 of the most representative B-cell (n = 203), T-cell (n = 30), and myeloid (n = 134) neoplasias, as well as 37 samples from different cell types of the hematopoietic system. Using appropriate controls of B-, T-, or myeloid cellular origin, we identified a total of 220 genes hypermethylated in at least one HN entity. In general, promoter hypermethylation was more frequent in lymphoid malignancies than in myeloid malignancies, being germinal center mature B-cell lymphomas as well as B and T precursor lymphoid neoplasias those entities with highest frequency of gene-associated DNA hypermethylation. We also observed a significant correlation between the number of hypermethylated and hypomethylated genes in several mature B-cell neoplasias, but not in precursor B- and T-cell leukemias. Most of the genes becoming hypermethylated contained promoters with high CpG content, and a significant fraction of them are targets of the polycomb repressor complex. Interestingly, T-cell prolymphocytic leukemias show low levels of DNA hypermethylation and a comparatively large number of hypomethylated genes, many of them showing an increased gene expression.

**Conclusions/Significance:**

We have characterized the DNA methylation profile of a wide range of different HNs entities. As well as identifying genes showing aberrant DNA methylation in certain HN subtypes, we also detected six genes—DBC1, DIO3, FZD9, HS3ST2, MOS, and MYOD1—that were significantly hypermethylated in B-cell, T-cell, and myeloid malignancies. These might therefore play an important role in the development of different HNs.

## Introduction

Hematological neoplasms (HNs) comprise a highly heterogeneous group of diseases showing different genetic, transcriptional, phenotypical and clinical features [1]. It is widely accepted that the acquisition of genetic changes taking place at different stages of maturation of the hematopoietic lineages plays an essential role in the development of HNs [2], [3]. These alterations include irreversible changes in the DNA sequence, like mutations, translocations, deletions, amplifications, etc. that result in gene activation or inactivation. Epigenetic changes, which represent reversible modifications that affect gene expression without altering the DNA sequence itself, are also a hallmark of cancer [4], [5]. The best studied epigenetic change is the hypermethylation of tumor suppressor genes which is reported to be associated with gene inactivation [6]. DNA methylation changes have been frequently described in various subtypes of HNs [7], [8], [9]. Most epigenetic studies in HNs have focused on the analysis of few tumor suppressor genes and several recent publications have characterized the DNA methylome of HNs by microarray-based approaches [10], [11], [12], [13], [14], [15], [16], [17], [18], [19], [20] These reports focused only on one or few HN subtypes. Therefore, the aim of our study was to provide a comparative overview of the DNA methylome of a wide range of HNs, including tumors of B-cell, T-cell and myeloid origin.

## Materials and Methods

### Patient samples and controls

A total of 367 samples from patients affected with HNs were analyzed in the present study, covering 16 different entities of B-cell, T-cell and myeloid origin. These included 9 B-cell neoplasms: diffuse large B-cell lymphoma (DLBCL, n = 54), molecular Burkitt lymphoma (mBL, n = 18), intermediate lymphoma (INT, n = 16) with a gene expression profile between mBL and non-mBL (i.e. DLBCL) [16], follicular lymphoma (FL, n = 14), mantle cell lymphoma (MCL, n = 10), multiple myeloma (MM, n = 14), B-cell chronic lymphocytic leukemia (B-CLL, n = 25), mucosa-associated lymphoid tissue (MALT) lymphoma (n = 10) and precursor B-cell acute lymphoblastic leukemia (B-ALL, n = 42). Four T-cell neoplasia entities were included: precursor T-cell acute lymphoblastic leukemia (T-ALL, n = 18), sorted CD3-positive cells of T-cell prolymphocytic leukemia (T-PLL, n = 4), anaplastic large cell lymphoma (ALCL, n = 3) and peripheral T-cell lymphoma (PTCL, n = 5). Finally, three myeloid leukemia subtypes were also included in the analysis: acute myeloid leukemia (AML, n = 116), myelodysplastic syndrome/myeloproliferative syndrome (MDS/MPS, n = 13) and chronic myelogenous leukemia (CML, n = 5). Some of the above listed entities comprise several sub-entities based on genetic, transcriptional or morphological analyses. However, as the goal of this study is to provide a global overview of DNA methylation changes in HNs, these sub-entities were not considered in detail for the present analysis. Three separate publications published or in preparation which will rely partly on the same dataset provide a detailed analysis of different subtypes of mature aggressive B-cell lymphomas (i.e. DLBCL, mBL and INT) [16], B- and T-ALL (Agirre et al., in preparation), and AML (Alvarez et al., in preparation).

As control samples, we used eight tissues or cell types from the hematopoietic system. These included whole peripheral blood (WPB, n = 4), whole bone marrow (WBM, n = 4), peripheral blood lymphocytes (PBL, n = 7), CD34-positive cells from BM (n = 4), CD3-positive T-cells from PB (n = 5), CD19-positive B-cells from PB (n = 5), germinal center B-cells (GCB, n = 2) as well as B-cell lymphoblastoid cell lines (LBL, n = 6) [16].

### DNA methylation profiling using universal BeadArrays

Microarray-based DNA methylation profiling was performed on all 367 hematological neoplasms and the 37 control samples with the GoldenGate Methylation Cancer Panel I (Illumina, Inc.). 247 samples were processed at the Illumina Headquarters (San Diego, CA) and 157 at the Spanish National Cancer Center (CNIO, Madrid, Spain). The panel is developed to assay 1505 CpG sites selected from 807 genes, which include oncogenes and tumor suppressor genes, previously reported differentially methylated or differentially expressed genes, imprinted genes, genes involved in various signaling pathways, and genes responsible for DNA repair, cell cycle control, metastasis, differentiation and apoptosis.

Methylation assay was performed as described previously [21]. Briefly, for each CpG site, four probes were designed: two allele-specific oligos (ASO) and two locus-specific oligos (LSO). Each ASO-LSO oligo pair corresponded to either the methylated or unmethylated state of the CpG site. Bisulfite conversion of DNA samples was done using the EZ DNA methylation kit (Zymo Research, Orange, CA). After bisulfite treatment, the remaining assay steps were identical to the GoldenGate genotyping assay [22] using Illumina-supplied reagents and conditions. The array hybridization was conducted under a temperature gradient program, and arrays were imaged using a BeadArray Reader (Illumina Inc.). Image processing and intensity data extraction software were performed as described previously [23], [24]. Each methylation data point is represented by fluorescent signals from the M (methylated) and U (unmethylated) alleles. Background intensity computed from a set of negative controls was subtracted from each analytical data point. The ratio of fluorescent signals was then computed from the two alleles according to the following formula:
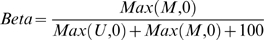
(1.1)


The beta value is a quantitative measure of DNA methylation levels of specific CpGs, and ranges from 0 for completely unmethylated to 1 for completely methylated.

The high reproducibility of the GoldenGate Methylation Cancer Panel I (mean coefficient of determination (R^2^) = 0.99) has been demonstrated previously [16].

Before analyzing the methylation data, we excluded possible sources of biological and technical biases that could alter the results. A known biological factor is that one copy of chromosome X is methylated in women and therefore, to avoid a gender-specific bias, all 84 CpGs on chromosome X were excluded from further analyses. Additionally, as the microarray experiments were run in two different labs using different BeadArray versions of the GoldenGate Methylation Cancer Panel I, we performed a differential methylation analysis to identify possible technical biases. We identified a total of 11 CpGs differentially methylated between the laboratories/platforms (p-value <0.01 and difference between mean beta values >0.2). After excluding 84 gender-specific CpGs and 11 CpGs showing interlab (i.e. interarray version) differential methylation, a total of 1410 CpGs from 767 genes entered further statistical analyses.

All DNA methylation data are available as Table S1, Table S2, and Table S3). All data is MIAME compliant as detailed on the MGED Society website http://www.mged.org/Workgroups/MIAME/miame.html.

### Hierarchical cluster analysis and differential methylation analysis

Hierarchical clustering was performed on all 367 cases and 30 controls using the Cluster Analysis tool of the BeadStudio software (version 3). Since PBLs contain a mixture of B and T cells, they were not used as control samples either for B cell or for T cell neoplasms.

Differential methylation analysis (DMA) was performed using the BeadStudio software (version 3). Different subtypes of B-cell, T-cell and myeloid tumors were compared with appropriate control samples (outlined in the results section). Two criteria were used to detect significantly differentially methylated CpGs. First, a false discovery rate (FDR) below 0.01 (Mann-Whitney U test) and a mean DNA methylation (Beta) values between tumors and controls showing a difference of at least 0.3. The statistical power of the Mann-Whitney U test was low for some comparisons due to small sample sizes. Therefore, a second criterion was used in which clear differential methylation was defined as a mean Beta values between tumors and controls showing a difference of at least 0.5. A CpG was then classified as differentially methylated if any, or both, criteria were met.

### Venn diagrams

To compare lists of genes differentially methylated in B-cell, T-cell and myeloid tumors, Venn diagrams were performed using the GeneVenn software developed at the University of Southern Mississippi (http://mcbc.usm.edu/genevenn/) [25].

### Correlation between DNA hypermethylation and hypomethylation

Pearson correlation coefficients and scatter plots were used to study the association between de novo gain and loss of gene-associated DNA methylation in different subtypes of hematological tumors (SPSS, version 15.0).

### Association between DNA hypomethylation and gene expression in T-PLL

Gene expression values of 66 tags from 39 hypomethylated genes in CD3-positive cells from five T-PLL cases (including two T-PLLs studied herein) were compared with eight normal CD3-positive samples from peripheral blood using data generated with the Affymetrix U133A array [26] (raw expression data has been deposited in a MIAME compliant format in the GEO database, accession number GSE5788). A fold change (in log2 scale) was calculated between the mean of gene expression data per tag in T-PLLs and T-cell controls.

### Enrichment for polycomb repressor complex 2 (PRC2) marks and promoter classes in differentially methylated genes

Proportions of PRC2 target genes and promoter classes in genes differentially methylated in various HNs (only those with at least eight differentially methylated genes were included) and all genes analyzed with the Illumina BeadArray were compared using the Fisher's exact test (SPSS, version 15.0). A genome-wide mapping of PRC2 genes in embryonic stem cells was available as supplemental material of the study by Lee et al. [27]. To analyze whether promoter regions of differentially methylated genes showed different CpG compositions, we used a recently described classification into promoters with high (HCP), intermediate (ICP) and low (LCP) CpG content [28].

## Results

### DNA methylation profiling of different control samples from the hematopoietic system

As DNA methylation patterns are tissue and cell type specific [29], the application of proper control samples is a main issue to detect de novo DNA methylation changes in tumor samples. To identify suitable controls for HNs of B-cell, T-cell and myeloid origin, we generated DNA methylation profiles of eight tissues or cell types from the hematopoietic system. As mentioned in the materials section, these included 37 samples from eight tissues or cell types of the hematopoietic system: WPB (n = 4), WBM (n = 4), PBL (n = 7), CD34-positive cells (n = 4), CD3-positive T-cells (n = 5), CD19-positive B-cells (n = 5), GCB cells, (n = 2) and LBL cell lines (n = 6).

**Figure 1:**
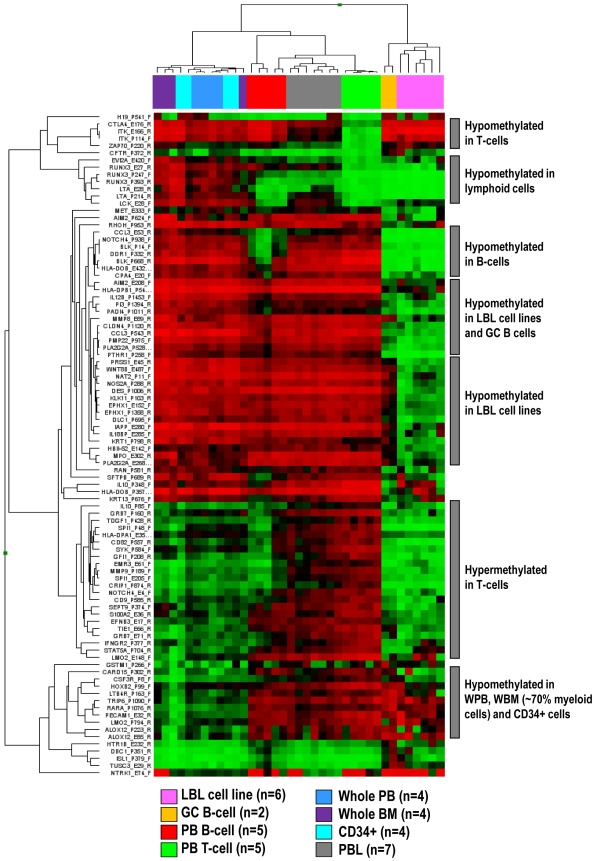
Hierarchical cluster analysis of CpGs differentially methylated in distinct non-malignant hematopoietic cell types.

A hierarchical cluster analysis of 1410 CpGs (from 767 genes) entering the statistical analyses showed that different control samples display differential DNA methylation patterns. We then compared the mean per CpG in each group with the mean per CpG in each of the other groups and identified genes showing differential methylation (defined as a difference of mean Beta values above 0.5) (Figure 1). No significant differences were detected between whole PB, whole BM and CD34-positive cells, which clustered together. Interestingly, these three controls showed hypermethylation of genes like RUNX3 whereas in lymphoid cells, RUNX3 was unmethylated. Lymphoid cells derived from PB, like B-cells, T-cells and PBLs clustered together. T-cells showed a homogeneous epigenetic pattern with hypomethylation of genes expressed in T-cells like ITK, CTLA4 or ZAP70. DNA methylation profiles of B-cells like CD19-positive PB cells, GC B-cells and LBL cell lines were heterogeneous. B-cell specific genes like BLK were hypomethylated in all three control types, but a higher number of hypomethylated genes were detected only in GC B-cells and/or LBL cell lines. Expectedly, as PBLs mostly contain a mixture of B- and T-cells, their methylation profile was in between that obtained for B- and T-cells.

According to these results, WPB, WBM and CD34-positive cells were used as controls for myeloid neoplasms and CD3-positive T-cells as controls for T-cell neoplasms. Considering the heterogeneous nature of the nine subtypes of B-cell neoplasms analyzed herein, we decided to group CD19-positive cells, GCB cells and LBL cell lines as controls. As PBLs contain a mixture of B and T cells, they were not used as control samples either for B cell or for T cell neoplasms.

### Global DNA methylation profiles in different subtypes of hematopoietic neoplasms

**Figure 2:**
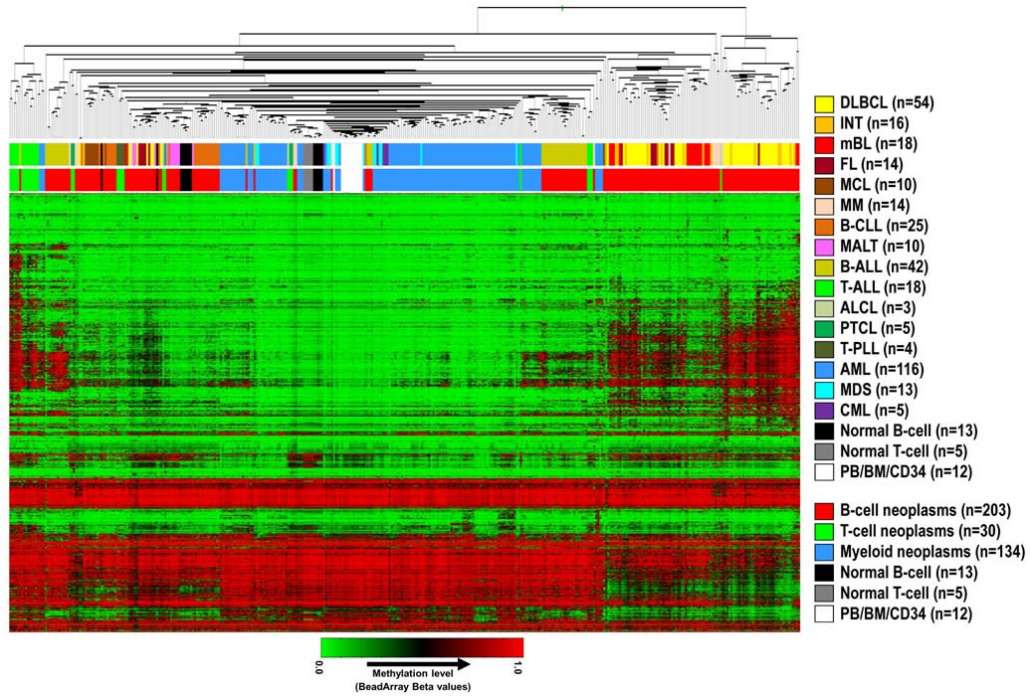
Hierarchical cluster analysis of DNA methylation data obtained from 367 HNs and 30 control samples. The upper bar below the dendrogram points to the specific HN subtype whereas the lower bar points to a classification of HNs according to the cell of origin (i.e. B-cell, T-cell, or myeloid cell). DLBCL: diffuse large B-cell lymphoma, INT: intermediate lymphoma, mBL: molecular Burkitt lymphoma, FL: follicular lymphoma, MCL: mantle cell lymphoma, MM: multiple myeloma, B-CLL: B-cell chronic lymphocytic leukemia, MALT: mucosa-associated lymphoid tissue lymphoma, B-ALL: precursor B-cell acute lymphoblastic leukemia, T-ALL: precursor T-cell acute lymphoblastic leukemia, ALCL: anaplastic large cell lymphoma, PTCL: peripheral T-cell lymphoma, T-PLL: T-cell prolymphocytic leukemia, AML: acute myeloid leukemia, MDS: myelodysplastic syndrome, CML: chronic myelogenous leukemia, PB: peripheral blood, BM: bone marrow, CD34: CD34-positive cells.

**Figure 3:**
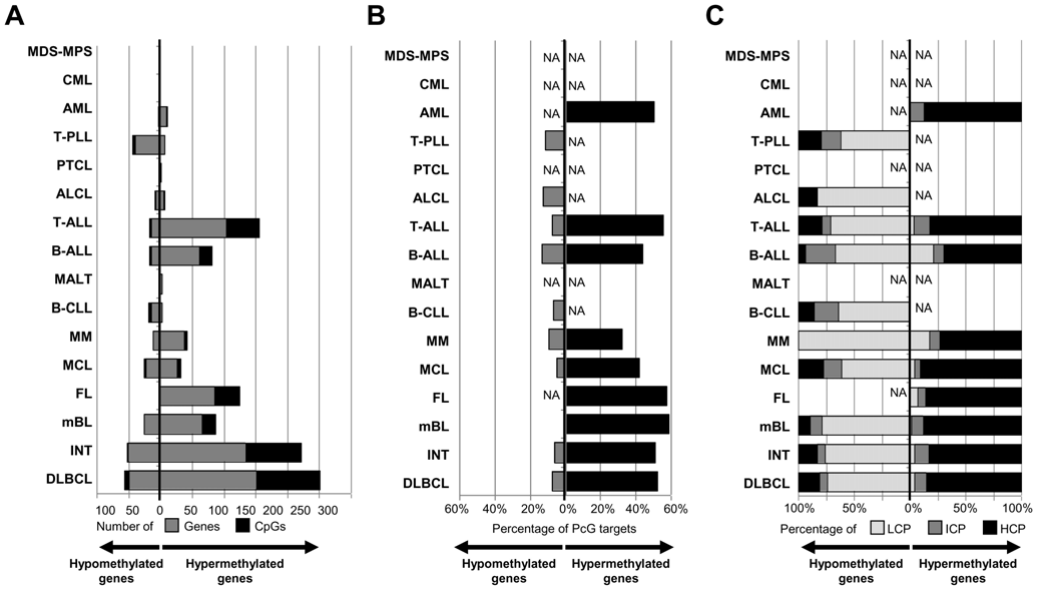
Bar plot of the different HNs under study showing for each entity. (A) The number of genes/CpGs hypermethylated or hypomethylated, (B) the percentage of differentially methylated genes marked by the PRC2 in ESCs, and (C) the percentage of differentially methylated genes showing distinct promoter subtypes according to their CpG content. LCP: low CpG content, ICP: intermediate CpG content, HCP: high CpG content.

A hierarchical cluster analysis of methylation values of 1410 CpGs in 367 hematological malignancies and 30 control samples (excluding PBL samples, which are composed of both B- and T-cells) is shown in Figure 2. Lymphoid malignancies were generally associated with higher levels of de novo DNA methylation than myeloid malignancies, which were mostly unmethylated and clustered together with normal control samples (Figure 2 and Figure 3A). Interestingly, high levels of de novo DNA methylation seemed to be prevalent especially in germinal center B-cell derived lymphomas like DLBCL, INT, mBL and FL, and lymphoid precursor cell derived tumors like B-lineage ALL (B-ALL) and T-lineage ALL (T-ALL).

The hierarchical cluster analysis in Figure 2 indicates that although some entities tend to cluster together (e.g. myeloid tumors or T-PLL), DNA methylation is heterogeneous within HN subtypes, especially in lymphoid tumors.

Separate cluster analyses of B-cell, T-cell and myeloid neoplasms with their respective controls are shown in Figure S1, Figure S2, and Figure S3.

### Detection of genes acquiring de novo DNA methylation in different subtypes of hematological neoplasms

**Figure 4:**
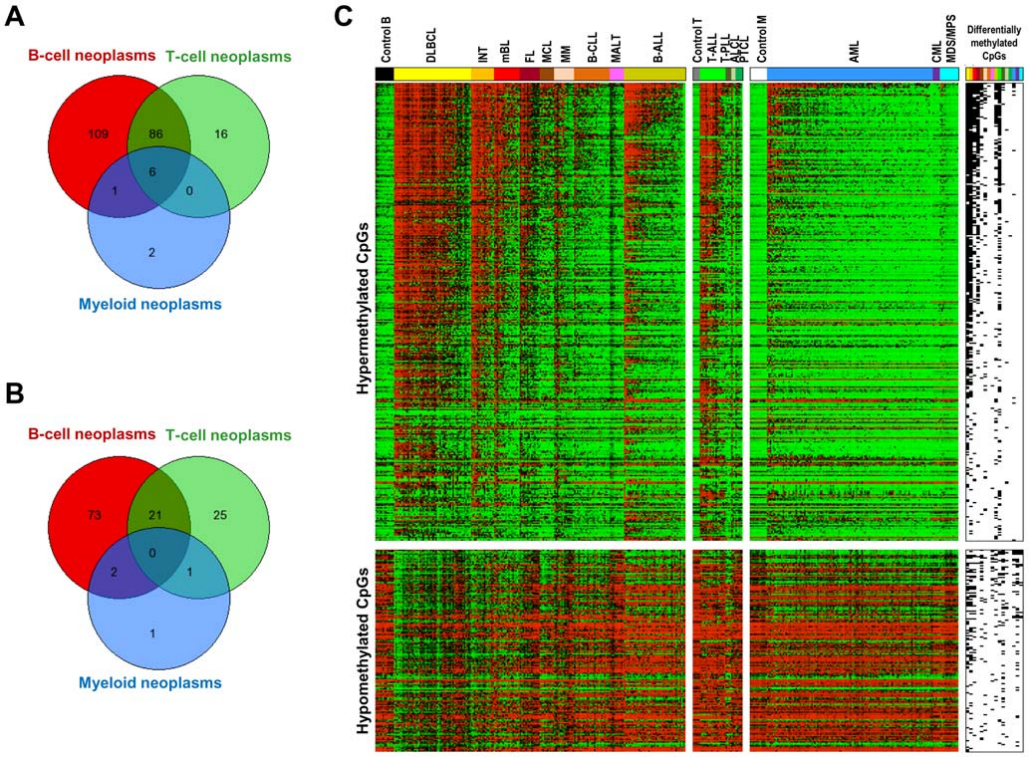
Venn diagrams showing the overlap of genes. (A) Hypermethylated or (B) hypomethylated in B-cell, T-cell and myeloid neoplasms. Only six genes were significantly hypermethylated in neoplasms of B-cell, T-cell and myeloid origin. We did not detect any gene hypomethylated in tumors of all three cellular origins. (C) Heatmap containing all the CpGs hypermethylated (above) or hypomethylated (below) in at least one HN subtype. CpGs are arranged in decreasing order of hypermethylation frequency. The presence of hypermethylated or hypomethylated CpGs in a given HN subtype is shown on the right of the heatmap (black: differentially methylated).

To identify genes de novo methylated in hematological neoplasms, differential methylation analysis (DMA) was performed comparing each tumor entity with the appropriate control samples. A summary of the results of the DMA is shown in Figure 3A and Figure 4A. A total of 354 CpGs (belonging to 220 genes) were shown to be hypermethylated in at least one entity. The heatmap on Figure 4C shows differentially methylated CpGs arranged according to the frequency of appearance in one or more HNs. The analyses shown in Figure 4 indicate that most of the hypermethylated genes are present in one or few malignancies and that a small number of genes are hypermethylated across HNs.

A list of de novo methylated genes in each tumor entity is shown in Table S4.

#### B-cell neoplasms

The DMA analyses in B-cell tumors showed that 326 CpGs (belonging to 202 genes) were statistically significantly hypermethylated in at least one subtype (Figure 3A, Figure 4A). Fifty-eight genes were shown to be de novo methylated in precursor B-ALLs. In mature B-cell tumors, a highly heterogeneous picture of DNA hypermethylation was obtained. Some entities like DLBCLs, INTs, mBLs and FLs were characterized by a high number of de novo methylated genes, which ranged from 87 in mBL to 251 in DLBCL. Other mature B-cell tumors like MCL or MM displayed an intermediate degree of de novo DNA methylation, showing 32 and 42 hypermethylated genes, respectively. Finally, B-CLLs and MALT lymphomas showed low levels of de novo DNA methylation, with only three genes being significantly methylated in each entity.

#### T-cell neoplasms

DMA comparing T-cell tumors versus T-cell control samples showed that 164 CpGs (108 genes) were hypermethylated in at least one T-cell neoplasm. The great majority of these genes were associated with T-ALL, in which 156 CpGs belonging to 103 genes were hypermethylated (Figure 3A). In contrast, the mature T-cell tumors studied displayed low levels of de novo DNA methylation, being one gene hypermethylated in PTCL, five in ALCL and six in T-PLL.

#### Myeloid neoplasms

A DMA in myeloid tumors showed that these entities were globally characterized by low levels of de novo DNA methylation (Figure 3A). In AML, a total of 11 CpGs (belonging to 9 genes) were shown to be hypermethylated. In MDS/MPS and CML, DMA did not detect any hypermethylated gene using the applied criteria.

#### Commonly hypermethylated genes

Of the 767 genes analyzed only six genes (0.8%) were commonly hypermethylated in HNs (Figure 4A), namely *DBC1*, *DIO3*, *FZD9*, *HS3ST2*, *MOS* and *MYOD1*. In contrast, the vast majority of the genes being hypermethylated in T-cell lymphomas were also found becoming hypermethylated in B-cell lymphomas (92 genes corresponding to 85% of all hypermethylated genes).

### Loss of gene-specific DNA methylation in hematological malignancies

DMA to detect genes suffering de novo loss of DNA methylation was performed comparing B-cell, T-cell and myeloid tumors with their respective control samples. These analyses indicate that gene-specific DNA hypomethylation, although less frequent than DNA hypermethylation, is also a common finding in hematological tumors (Figure 3A), at least for the 767 genes under analysis. The highest number of hypomethylated genes was found in B-cell neoplasms (96 genes), followed by T-cell (47 genes) and myeloid neoplasms (4 genes). However, none of these genes analyzed became hypomethylated in all entities (Figure 4B). A list of hypomethylated genes in each tumor entity is shown in Table S5.

### Correlation between de novo loss and gain of gene-specific DNA methylation in hematological malignancies

**Figure 5:**
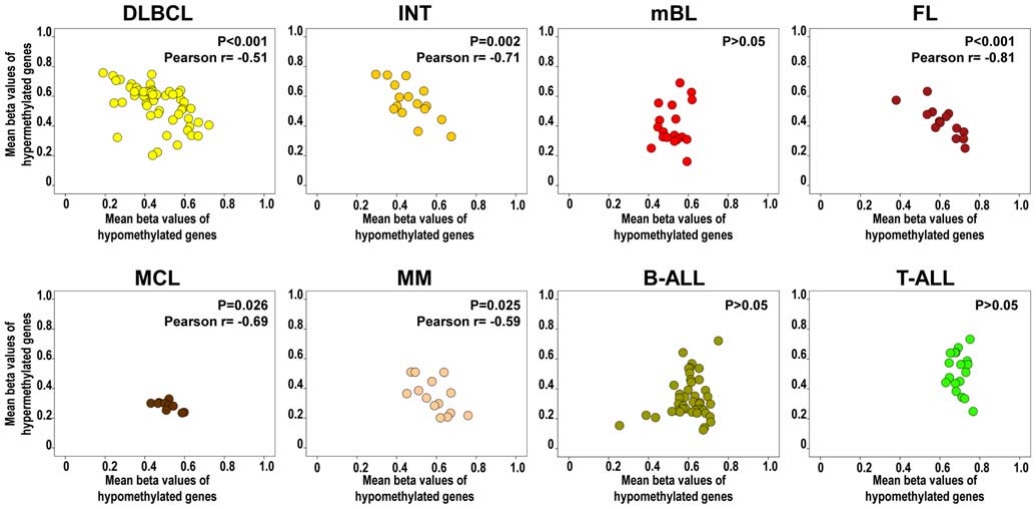
Scatter plots of mean methylation values of hypermethylated genes (Y axis) and hypomethylated genes (X axis) in HNs with more than 10 hypermethylated genes. This figure shows that there is a significant correlation between the level of DNA hypermethylation (increasing beta values) and DNA hypomethylation (decreasing beta values) in cases diagnosed with DLBCL, INT, FL, MCL and MM. In contrast, such phenomenon is not observed in mBL and precursor lymphoid leukemias like B-ALL and T-ALL.

Figure 3 and Figure 4 suggest that some tumors with a large number of hypermethylated genes were accompanied by increased levels of hypomethylated genes. To investigate whether gene-specific DNA hypermethylation was associated with DNA hypomethylation, we calculated Pearson correlation coefficients and plotted the average methylation values per case for all hyper- and hypomethylated genes in HNs with at least 10 hypermethylated genes. The results indicate that DNA hypermethylation significantly correlates with DNA hypomethylation in most mature B-cell tumors, i.e. cases with higher levels of hypermethylation also show higher levels of hypomethylation. In contrast, B- and T-ALL, although they are heavily methylated, they do not show a concurrent de novo loss of DNA methylation (Figure 5).

### Association between PRC2 targets in stem cells and genes differentially methylated in hematological malignancies

We investigated whether genes showing significant differential methylation in HNs were among those repressed by the PRC2 in embryonic stem cells [27]. As shown in Figure 3B, between 32% and 59% of the genes suffering de novo methylation in HNs are PRC2 targets in embryonic stem cells. As compared with the 21% of PRC2 targets in all the genes studied with the array, this enrichment was statistically significant in all HNs studied but MM and AML (Table S6). In contrast, only 5 to 13% of the hypomethylated genes were PRC2 targets in embryonic stem cells. These findings indicate that, regardless of the cellular nature of the HN entity, de novo DNA methylation of a large proportion of genes seems to be mediated by members of the polycomb complex.

### The promoter regions of genes losing or acquiring DNA methylation show different CpG content

To investigate whether the promoter regions of the genes differentially methylated in different hematological tumors showed a specific CpG composition, we classified them into high (HCP), intermediate (ICP) and low (LCP) CpG content using a recent classification system [28]. In line with previous reports [16], our analysis demonstrated that genes acquiring de novo methylation in tumors are mostly characterized by promoters with high CpG content (Figure 3C). In contrast, the proportion of CpGs in gene promoters showing de novo loss of DNA methylation in hematological tumors is usually low (Figure 3C).

### Loss of gene-specific DNA methylation is more frequent than hypermethylation in T-PLL and correlates with increased gene expression

**Figure 6:**
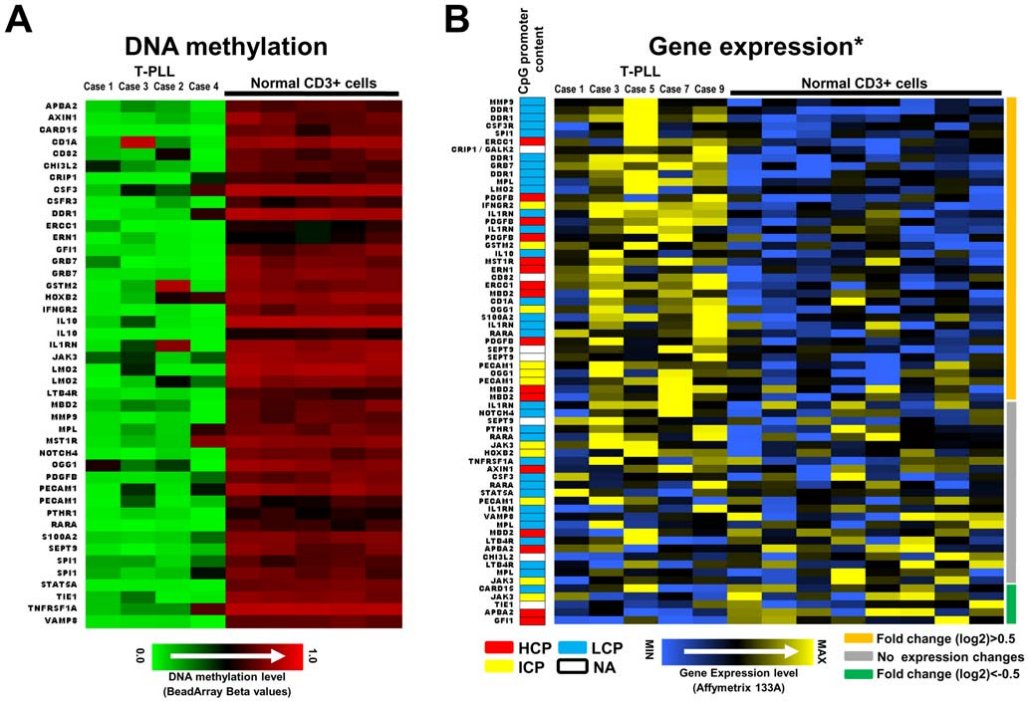
Most genes showing hypomethylation of promoter regions in T-PLL are associated with increased gene expression. (A) Genes hypomethylated in T-PLL as compared with CD3+ normal T cells. Genes are arranged alphabetically. (B) Gene expression of hypomethylated genes in T-PLL cases as compared to CD3+ normal T cells. Genes are arranged according to their differential expression in T-PLL vs. normal controls (most upregulated genes on the top). Genes showing at least a 0.5 fold increase (in log2 scale), no expression changes or a 0.5 fold decrease are marked with orange, grey or green lines, respectively, on the right of the heatmap. The CpG content of the promoter sequences of the genes presented is depicted as color code in a separate column (low CpG content (LCP) = blue, intermediate CpG content (ICP) = yellow, high CpG content (HCP) = red, white = data not available). Two of the T-PLL cases (case 1 and case 3) have been studied both by DNA methylation and gene expression profiling. The normal CD3+ T cells used for DNA methylation and gene expression profiling were different. * Gene expression data have been published before by Dürig et al. [26]

DNA hypermethylation is clearly more frequent than hypomethylation in HNs (Figure 3A). However T-PLL represents a striking exception to that observation. The four CD3+ tumor cells of the T-PLLs studied show a homogeneous DNA methylation profile characterized by multiple genes suffering de novo loss of DNA methylation (n = 44) and few hypermethylated genes (n = 6). Comparing microarray-based gene expression of hypomethylated genes in the CD3+ T-PLLs cells to CD3+ T-cell controls, we detected a total of 38 tags showing at least a 0.5 fold (in log2 scale) increase in gene expression in T-PLL vs T-cell controls. These include *PDGFB*, *MMP3*, *DDR1*, *IL1RN*, *IL10*, *CSF3R* or *SEPT9*, which predominantly contained promoters with low CpG content (LCP) (Figure 6B). In contrast, only 5 tags showed log2 fold change values below −0.5. The remaining 23 tags showed fold changes between −0.5 and 0.5. These results indicate that gene promoter hypomethylation is frequently associated with increased gene expression in T-PLL (Figure 6).

## Discussion

The main goal of this study was to provide a general overview of DNA methylation changes associated with the most common subtypes of HNs. These HNs were derived from different cell lineages of the hematological system, i.e. B-cells, T-cells and myeloid cells at different stages of differentiation. As DNA methylation profiles are tissue and cell type-specific [29], we initially analyzed methylation profiles of various cell types and tissues of the hematopoietic system to identify appropriate control samples for the different HNs under study. In this way, we eliminated cell lineage-specific biases and were able to detect tumor-specific DNA methylation changes.

Statistical analyses of the microarray data identified the presence of DNA methylation changes associated with the 16 different subtypes of HNs under study. Our data clearly indicate that DNA hypermethylation patterns are not homogenous across different HNs. At the global level, we could observe that lymphoid neoplasms generally showed a higher number of de novo methylated genes than myeloid neoplasms. Interestingly, most myeloid neoplasms only showed low levels of aberrant DNA methylation and clustered together with the normal control samples (Figure 2).

Using the defined statistical criteria (see methods section), we detected DNA methylation changes characteristic for the HN entities under study (Table S4 and Table S5). Overall, we have identified 343 genes differentially methylated in at least one entity. Of these genes 220 become hypermethylated while 123 become hypomethylated. In line with previous reports in solid tumors [30], [31], [32], [33] and mature aggressive B-cell lymphomas [16], a high proportion of genes de novo methylated in various HN subtypes were repressed by PRC2 in embryonic stem cells.

As well as genes predominantly methylated in certain HN subtypes, we also identified six genes, i.e. *DBC1*, *DIO3*, *FZD9*, *HS3ST2*, *MOS* and *MYOD1*, that were significantly hypermethylated in B-cell, T-cell and myeloid malignancies. Hypermethylation of DBC1, HS3ST2 and MYOD1 has been reported before not only in HNs [12], [18], [34], [35], [36], [37] but also in solid tumors [38], [39], [40]. Therefore, silencing of these genes by hypermethylation seems to be a frequent pathomechanism associated with most human cancers.

FZD9 (Frizzled homolog 9) is a receptor for WNT2 in the WNT pathway, from which several members have been shown to be epigenetically deregulated in HNs [41]. De novo methylation of *FZD9* has been recently reported to be a frequent event in AML samples and an independent predictor of prognosis for patients with MDS/AML [12]. In our series, *FZD9* was shown to be significatively methylated in AML, but not in MDS or CML (Table S4).

The *MOS* (V-mos Moloney murine sarcoma viral oncogene homolog) gene has been previously identified as hypermethylated in ALL but not in AML [17]. In our series, although *MOS* is more frequently hypermethylated in ALL (mean beta value = 0.64) than in AML (mean beta value = 0.48), it also shows significant hypermethylation in AML. Another recent study shows that *MOS* is hypermethylated in AMLs with silenced *CEPBA* [18], suggesting that *MOS* is hypermethylated only in some AML subtypes.

*DIO3* (Deiodinase, iodothyronine, type III) is located in the imprinted region in chromosome band 14q32 and plays an essential role for regulation of thyroid hormone inactivation during embryological development. Recent data have shown that this gene is not expressed or expressed at low levels in hematological cells [42]. In spite of this lack of expression, our data indicate that the hypermethylated *DIO3* CpG site in HNs is unmethylated in controls. Recently, *DIO3* was found to be methylated in AMLs with *CEBPA* silencing [18].

Remarkably, we observed that precursor B- and T-cell neoplasms, i.e. precursor B-ALL and T-ALL, as well as mature B-cell neoplasms with features of germinal center like DLBCL, INT, mBL and FL are characterized by a large number of hypermethylated genes. In contrast, other lymphoid malignancies like MCL, MM, B-CLL, MALT, ALCL and T-PLL show intermediate to low DNA hypermethylation. These data suggest that lymphoid tumors arising from precursor cells and germinal center cells tend to acquire higher levels of de novo DNA methylation. Specific physiological rearrangements of immunoglobulin (*IG*) and T-cell receptor (*TCR*) genes differentiate these two stages of lymphocyte development from other maturation stages. VDJ rearrangements of *IGH* and *TCR* genes take place in precursor B-cell and T cells in the bone marrow, respectively, whereas class switch recombination of the *IGH* locus takes place in germinal centers of secondary lymphoid organs like lymph nodes. A possible link between physiological genetic rearrangements, lymphoid developmental stages and DNA methylation is derived from reports indicating that the polycomb protein EZH2 is involved in B-cell development and VDJ recombination [43], and that EZH2 is able to lead to DNA methylation through recruitment of DNA methyltransferases (DNMTs) [44]. Furthermore, immunohistochemical studies have revealed that EZH2 levels are higher in precursor T-cells than in resting mature T-cells [45], and that in the germinal center, proliferating centroblasts express EZH2 whereas non-proliferating centrocytes and naïve B cells do not [46]. In this scenario, we hypothesize that the features of the cell that initiates development of lymphoid neoplasms or the stage at which the tumor is frozen might shape the acquisition of DNA methylation changes. We postulate that germinal center lymphomas like DLBCL, INT, mBL and FL and precursor lymphoid neoplasms like pre B-ALL and T-ALL are initiated or frozen in developmental stages with proliferating cells and high EZH2 expression. Thus, eventual recruitment of DNMTs by EZH2 can then lead to a high level of aberrant DNA methylation in precursor and germinal center lymphoid neoplasms. The fact that neoplasms derived from myeloid cells, that do not suffer such physiological rearrangements, display low levels of aberrant DNA hypermethylation might support our hypothesis.

Another interesting finding derived from our data is that DNA hypermethylation is accompanied in mature B-cell lymphomas (with the exception of mBL) by gene-specific DNA hypomethylation (Figure 3, Figure 4, and Figure 5). In fact, cases with a large number of hypermethylated genes concurrently show large numbers of hypomethylated genes. In contrast, this effect is not observed in precursor lymphoid neoplasms like T-ALL or B-ALL (Figure 5). It has been widely reported in the literature that tumors show global hypomethylation of DNA repeats and local hypermethylation of gene promoters [4]. However, gene hypomethylation has received less attention [47], [48], although our data indicate that it is also a frequent phenomenon in cancer, at least in HNs. To our knowledge, correlation between levels of gene hyper- and hypomethylation has not been reported before.

In general, DNA hypermethylation is more frequent than DNA hypomethylation in HNs (Figure 3A). However, T-PLL, a rare mature T-cell neoplasm, represents an exception to this general rule. This neoplasm is characterized by a large number of hypomethylated genes and few hypermethylation events. DNA hypomethylation in T-PLL, and HNs in general, frequently targets promoters with low CpG content (Figure 3C), whose methylation has been reported to be independent from gene expression [28]. In the T-PLLs described here, however, most hypomethylated genes were associated with increased gene expression as compared to normal T cells (Figure 6) [26]. Some of these genes have been shown to be involved in tumorigenesis. For instance, interleukin 10 (IL10) acts as a negative regulator in numerous immunmodulatory processes [49]. Furthermore, IL10 expression has been correlated with tumor progression being of prognostic value in distinct entities [50], [51], [52]. Additionally, we here could show that both the DNA methylation and gene expression profile of another key player of the interleukin signaling pathway, interleukin 1 receptor antagonist (IL1RN), is altered in T-PLL. IL1RN has also been shown to be involved in tumorigenesis in numerous tumor entities [53], [54], [55], [56].

DDR1 belongs to the family of tyrosine kinases with high impact on cell transformation in distinct solid tumors [57], [58], [59]. Since DDR1 inhibition significantly increases chemosensitivity, this protein has been suggested to be a promising therapeutic target [60].

The platelet-derived growth factor (PDGFB) which becomes also hypomethylated and differentially expressed in T-PLL is involved in mitogenesis of mesenchymal cells and a known oncogene [61], [62], [63].

Our findings not only provide new insights into the biology of HNs but have potential therapeutic implications. DNA methylation inhibitors like Decitabine and 5-Azacitidine are currently used in clinical studies to treat patients with MDS and AML [64]. For instance, results from a phase III study have indicated that treatment of patients with higher risk MDS with 5-azacitidine results in significant improvement in overall survival [65]. However, considering that MDS and AML are among those HNs with less number of genes suffering de novo DNA methylation, we wonder whether part of the therapeutic effect of DNMT inhibitors is not due to demethylation of hypermethylated genes but rather a different mechanism, e.g. a cytotoxic effect. In any case, if DNMT inhibitors do base their therapeutic effect on demethylation of hypermethylated genes, then HNs with higher levels of DNA hypermethylation, like germinal center B-cell lymphomas and precursor lymphoid neoplasms, might theoretically be better targets for such drugs. DNA methylation profiling could be then a useful approach to monitor the association between epigenetic response and clinical response and to stratify patients for treatment with demethylating agents.

## Supporting Information

Figure S1 Hierarchical cluster analysis of DNA methylation data obtained from B-cell neoplasms. Hierarchical cluster analysis of DNA methylation data obtained from 203 B-cell neoplasms and 13 control samples. The bar below the dendrogram points to the specific subtype. DLBCL: diffuse large B-cell lymphoma, INT: intermediate lymphoma, mBL: molecular Burkitt lymphoma, FL: follicular lymphoma, MCL: mantle cell lymphoma, MM: multiple myeloma, B-CLL: B-cell chronic lymphocytic leukemia, MALT: mucosa-associated lymphoid tissue lymphoma, B-ALL: precursor B-cell acute lymphoblastic leukemia, LBL: lymphoblastoid cell line, GCB: germinal center B-cells, PB-B: peripheral blood B-cells.
(2.76 MB TIF)

Figure S2 Hierarchical cluster analysis of DNA methylation data obtained from T-cell neoplasms. Hierarchical cluster analysis of DNA methylation data obtained from 30 T-cell neoplasms and 5 control samples. The bar below the dendrogram points to the specific subtype. T-ALL: precursor T-cell acute lymphoblastic leukemia, ALCL: anaplastic large cell lymphoma, PTCL: peripheral T-cell lymphoma, T-PLL: T-cell prolymphocytic leukemia.
(0.94 MB TIF)

Figure S3 Hierarchical cluster analysis of DNA methylation data obtained from myeloid neoplasms. Hierarchical cluster analysis of DNA methylation data obtained from 134 myeloid neoplasms and 12 control samples. The bar below the dendrogram points to the specific subtype. AML: acute myeloid leukemia, MDS: myelodysplastic syndrome, CML: chronic myelogenous leukemia, PB: peripheral blood, BM: bone marrow, CD34: CD34-positive cells.
(1.52 MB TIF)

Table S1 Raw data: methylation values.
(5.96 MB TXT)

Table S2 Raw data: Cy3-intensity.
(5.13 MB TXT)

Table S3 Raw data: Cy5-intensity.
(5.13 MB TXT)

Table S4 Hypermethylated genes per entity. This table summarizes genes becoming hypermethylated in hematological neoplasms.
(0.06 MB XLS)

Table S5 Hypomethylated genes per entity. This table summarizes genes becoming hypomethylated in hematological neoplasms.
(0.04 MB XLS)

Table S6 Percentage of hypermethylated genes targeted by the PRC2 in ESCs. This table presents the percentage of hypermethylated genes which become targeted by the polycomb repressor complex 2 (PRC2) in embryonal stem cells (ESCs) in distinct lymphoma entities.
(0.03 MB DOC)
