# Supplementary figures and images for: A Comprehensive Microarray-Based DNA Methylation Study of 367 Hematological Neoplasms
Source: PLoS One. 2009 Sep 11;4(9):e6986. doi: 10.1371/journal.pone.0006986 (PMC2737286; doi:10.1371/journal.pone.0006986)

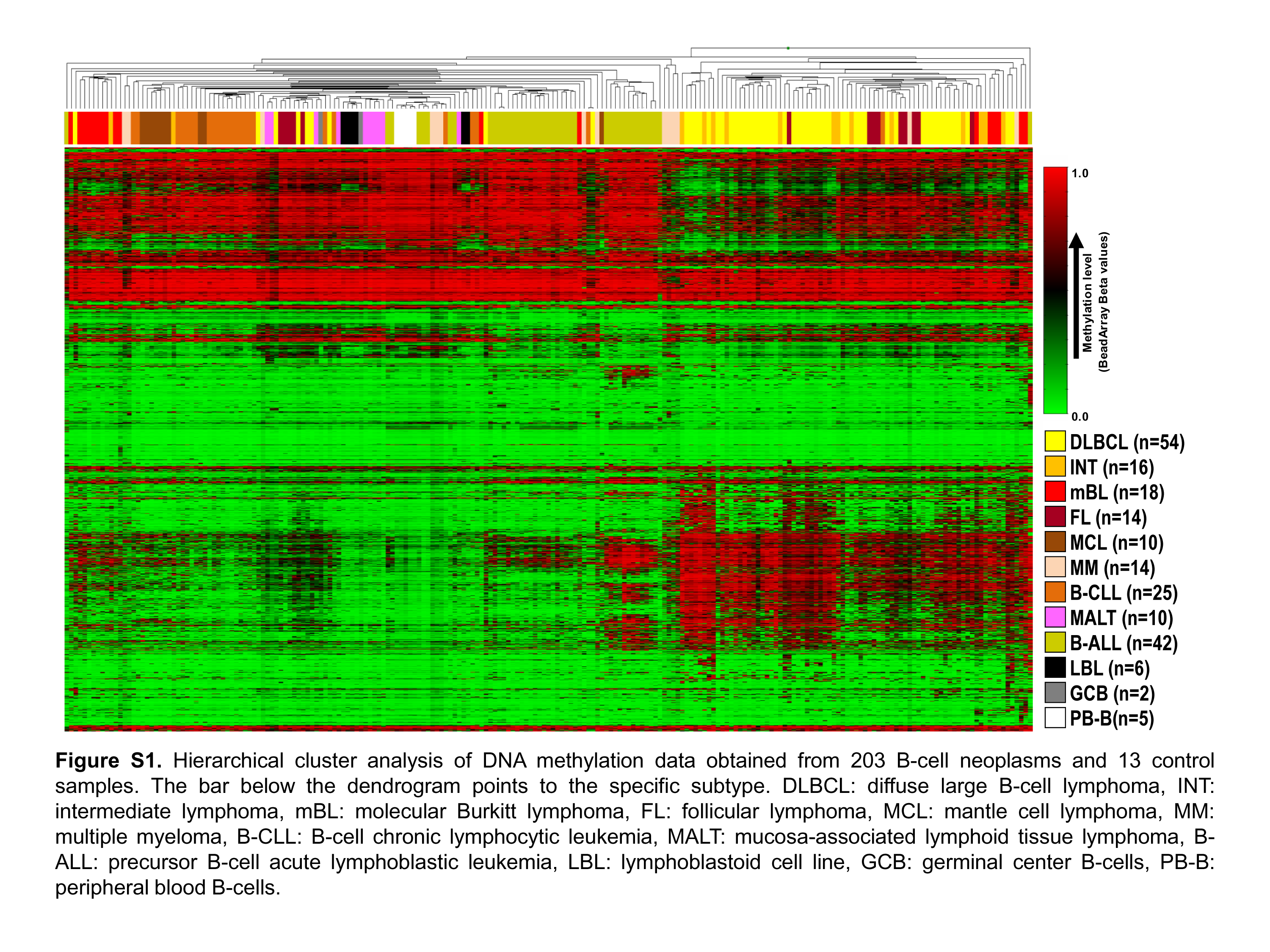

Supplement: Figure S1 — Hierarchical cluster analysis of DNA methylation data obtained from B-cell neoplasms. Hierarchical cluster analysis of DNA methylation data obtained from 203 B-cell neoplasms and 13 control samples. The bar below the dendrogram points to the specific subtype. DLBCL: diffuse large B-cell lymphoma, INT: intermediate lymphoma, mBL: molecular Burkitt lymphoma, FL: follicular lymphoma, MCL: mantle cell lymphoma, MM: multiple myeloma, B-CLL: B-cell chronic lymphocytic leukemia, MALT: mucosa-associated lymphoid tissue lymphoma, B-ALL: precursor B-cell acute lymphoblastic leukemia, LBL: lymphoblastoid cell line, GCB: germinal center B-cells, PB-B: peripheral blood B-cells. (2.76 MB TIF) [file pone.0006986.s001.tif]

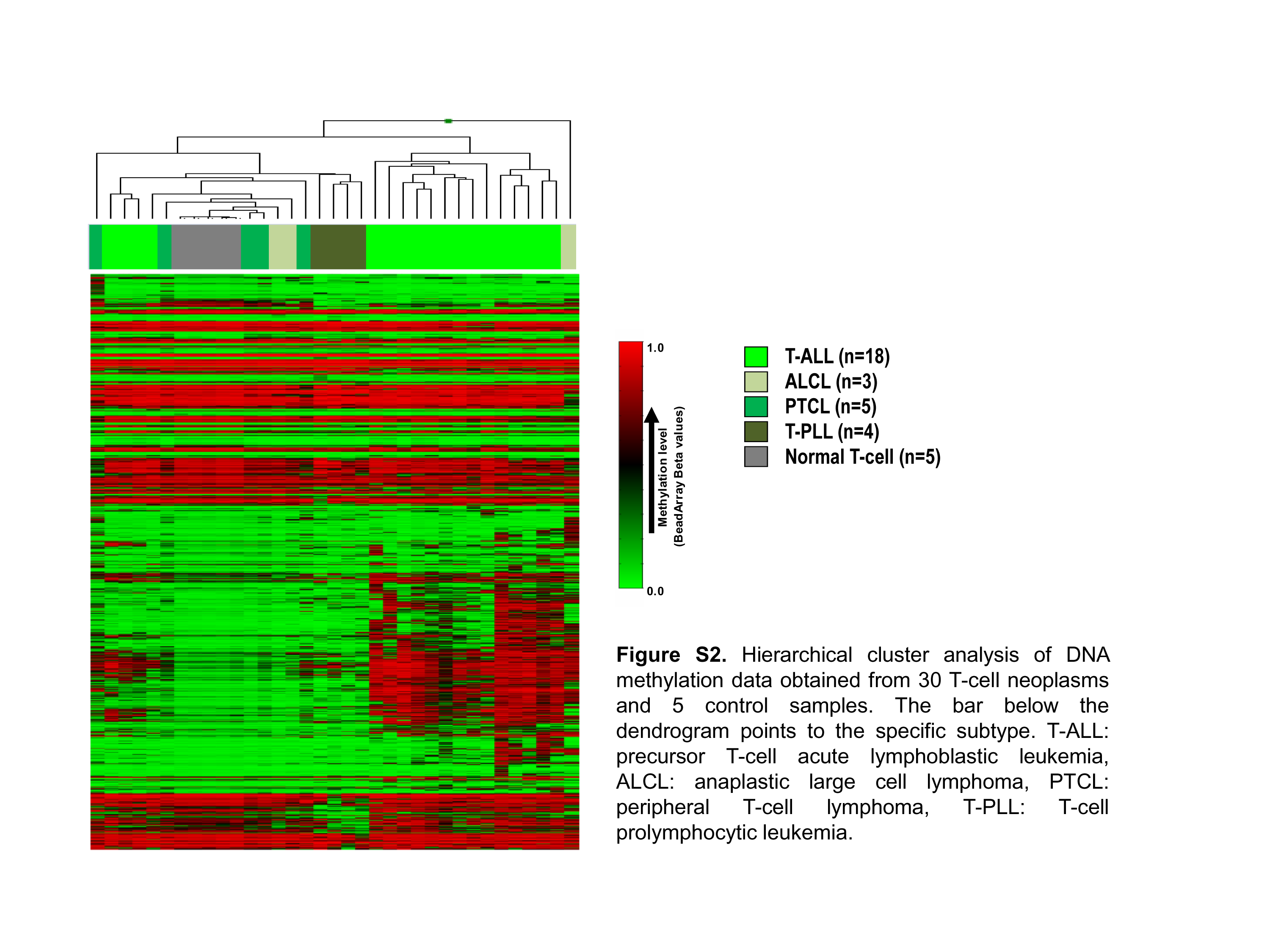

Supplement: Figure S2 — Hierarchical cluster analysis of DNA methylation data obtained from T-cell neoplasms. Hierarchical cluster analysis of DNA methylation data obtained from 30 T-cell neoplasms and 5 control samples. The bar below the dendrogram points to the specific subtype. T-ALL: precursor T-cell acute lymphoblastic leukemia, ALCL: anaplastic large cell lymphoma, PTCL: peripheral T-cell lymphoma, T-PLL: T-cell prolymphocytic leukemia. (0.94 MB TIF) [file pone.0006986.s002.tif]

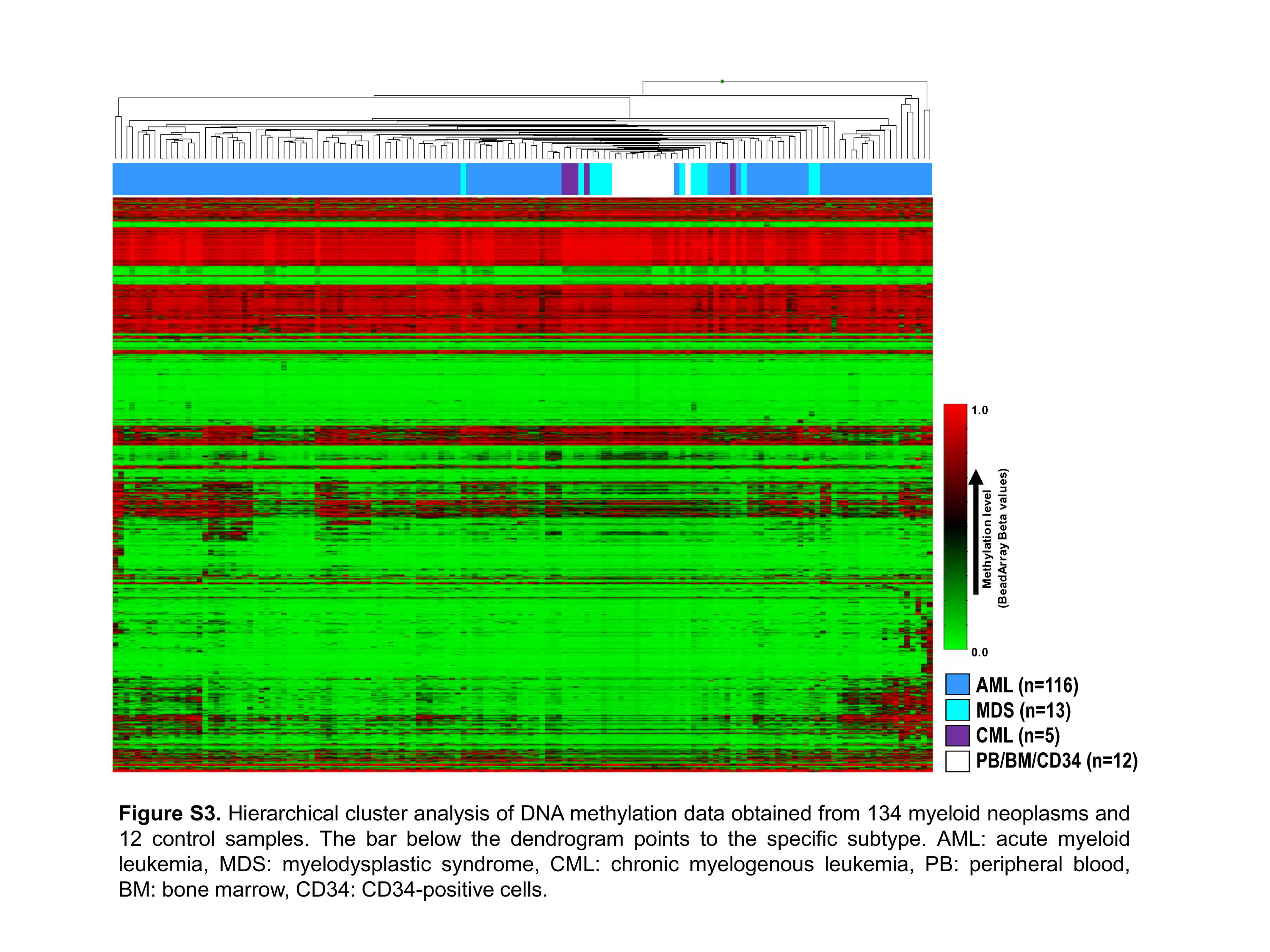

Supplement: Figure S3 — Hierarchical cluster analysis of DNA methylation data obtained from myeloid neoplasms. Hierarchical cluster analysis of DNA methylation data obtained from 134 myeloid neoplasms and 12 control samples. The bar below the dendrogram points to the specific subtype. AML: acute myeloid leukemia, MDS: myelodysplastic syndrome, CML: chronic myelogenous leukemia, PB: peripheral blood, BM: bone marrow, CD34: CD34-positive cells. (1.52 MB TIF) [file pone.0006986.s003.tif]
