# Supplementary material for: A Comprehensive Microarray-Based DNA Methylation Study of 367 Hematological Neoplasms
Source: PLoS One. 2009 Sep 11;4(9):e6986. doi: 10.1371/journal.pone.0006986 (PMC2737286; doi:10.1371/journal.pone.0006986)
Supplement: Table S6 — Percentage of hypermethylated genes targeted by the PRC2 in ESCs. This table presents the percentage of hypermethylated genes which become targeted by the polycomb repressor complex 2 (PRC2) in embryonal stem cells (ESCs) in distinct lymphoma entities. (0.03 MB DOC) [file pone.0006986.s009.doc]

**Table S6.** Percentage of hypermethylated genes targeted by the

PRC2 in ESCs [25]

| **Entity** | **Percentage of PRC2 targets**  **in ESCs** | | **Fisher’s exact test (p value)^1^** | |
| --- | --- | --- | --- | --- |
| DLBCL | 52.5 | <0.001 ** | |  |
| INT | 50.8 | <0.001 ** | |  |
| mBL | 58.7 | <0.001 ** | |  |
| FL | 57.3 | <0.001 ** | |  |
| MCL | 41.7 | 0.022 * | |  |
| MM | 32.4 | 0.131 | |  |
| B-ALL | 43.1 | 0.001 ** | |  |
| T-ALL | 55.7 | <0.001 ** | |  |
| AML | 50 | 0.07 | |  |

^1^As compared to the 21% of PRC2 target genes in all the analyzed genes
